# Do alterations in muscle strength, flexibility, range of motion, and alignment predict lower extremity injury in runners: a systematic review

**DOI:** 10.1186/s40945-019-0054-7

**Published:** 2019-02-12

**Authors:** Shefali M. Christopher, Jeremy McCullough, Suzanne J. Snodgrass, Chad Cook

**Affiliations:** 1 Department of physical therapy Education, Elon University, Elon, NC 27244 USA; 2 School of Health Sciences, The University of Newcastle, Callaghan, Australia; 3 Pivot Physical Therapy, Culpeper, VA 22701 USA; 4 Division of Physical Therapy, Duke University, 2200 W. Main Street, Durham, NC 27705 USA

**Keywords:** Running, Examination, Injury

## Abstract

**Background:**

Injury is common in running and seen to impact up to 94% of recreational runners. Clinicians often use alterations from normal musculoskeletal clinical assessments to assess for risk of injury, but it is unclear if these assessments are associated with future injury.

**Objectives:**

To identify alterations in muscle strength, flexibility, range of motion, and alignment that may predict lower extremity injury in runners.

**Methods:**

Articles were selected following a comprehensive search of PubMed, Embase, CINAHL, and SPORTDiscus from database inception to May 2018. Included articles were prospective cohort studies, which specifically analyzed musculoskeletal impairments associated with future running-related injury. Two authors extracted study data, assessed the methodological quality of each study using the Critical Appraisal Tool and assessed the overall quality using the GRADE approach.

**Results:**

Seven articles met the inclusion criteria. There was very low quality of evidence for the 7 identified clinical assessment alteration categories. Strong hip abductors were significantly associated with running-related injury in one study. Increased hip external-to-internal rotation strength and decreased hip internal range of motion were protective for running injury, each in one study. Decreased navicular drop in females had a protective effect for running-related injury in one study.

**Conclusions:**

Due to very low quality of evidence for each assessment, confounders present within the studies, a limited number of studies, different measurement methods among studies, measurement variability within clinical assessments, inconsistent definitions of injury and runner, different statistical modeling, and study bias, caution is suggested in interpreting these results.

## Background

Injury in runners is common, affecting 19.4 to 94.4% of runners annually [1, 2]. A high incidence of lower extremity running injuries such as Achilles tendinopathy, anterior and/or lateral knee pain, hamstring injury, stress fractures, or medial tibial stress syndrome, is reported commonly in the scientific literature [1, 3]. Despite widespread research on running injuries and their treatment, there are few long-term strategies or guidelines for preventing injuries in runners [4]. Alterations in objective musculoskeletal clinical assessments that predict whether a runner is at risk of injury might potentially form the basis of long-term prevention strategies.

A method for identifying those at risk for future running-related injuries is necessary in clinical or community wellness settings. Recently, researchers have focused on developing models to predict running-related injury (RRI) by examining the interaction of factors such as training related characteristics (i.e. work load) [5] and acute to chronic workload ratios (i.e. changes in weekly running distance) [6, 7]. Several studies [8–15] have investigated running gait and formally evaluated kinematic and kinetic factors that may predict or differentiate an injured runner from an uninjured runner. However, kinematic measures used in laboratories are not readily transferable to clinical practice, as they require complex equipment such as force plates and motion analysis systems.

In practice, clinicians use objective assessments to determine alterations in muscle strength, muscle flexibility, joint range of motion, and alignment during evaluation of runners. Clinicians use results of these tests to explain RRI to patients [16] as these assessments have been hypothesized to be associated with running injuries [17–19]. They often rely on the results of single studies reporting individual tests as well as studies that use cross sectional designs. To our knowledge, alterations in objective musculoskeletal clinical assessments have not been formally investigated for their ability to predict injury in runners in a systematic review. Therefore, the objective of this review is to identify alterations in muscle strength, flexibility, joint range of motion, and alignment that may predict lower extremity injury in runners in order to improve future statistical modeling for injury risks in runners. Syntheses of clinical assessments’ utility may assist clinicians who commonly use stand-alone findings from single cross-sectional studies to evaluate risk in athletes.

## Methods

### Study design

This study used the *Preferred Reporting Items for Systematic Reviews and Meta-Analyses* (PRISMA) statement during the search and reporting phase of this systematic review [20]. The systematic review was also registered with PROSPERO International prospective register of systematic reviews (CRD42016020087).

### Search strategy

PubMed, Cumulative Index of Nursing and Allied Health Literature (CINAHL), Embase, and SPORTDiscus databases were searched in consultation with a biomedical librarian to identify studies reporting the use of objective musculoskeletal clinical assessments predicting lower extremity injury in runners from database inception to May 2018. Keywords and standardized vocabulary (e.g. medical subject headings (MeSH) for PubMed) were combined with Boolean operators to build the searches. The search terms for PubMed are included in Appendix 1. The searches for CINAHL, Embase, and SPORTDiscus were built from the PubMed search using controlled vocabulary for each database. A detailed hand search involving references from the selected articles and gray literature was conducted, as computerized searches can occasionally omit relevant articles. Searches were limited to humans.

### Inclusion/exclusion criteria

We included only prospective cohort studies with longitudinal designs examining the relationship between musculoskeletal clinical assessments of the lower extremity assessed in a baseline cohort of runners who were uninjured and were followed over time to identify occurrence of an RRI. This inclusion criteria assisted our aim of predictive modeling, as the included studies “predict the output value for new observations given their input value” [21]. We only included studies that reported on strength of association (i.e., odds, hazard, or risks ratios in either bivariable or multivariable models) to assist predictive modelling. Odds ratio is used to compare the odds of an outcome when exposed to the variable of interest [22], hazard ratio measures the risk of complication given different event rates [23], and risk ratio measures risk of an event happening in one group compared to another group [24].

Running-related injury was operationally-defined in this review by at least one of the following: 1) diagnosed by a medical physician, athletic trainer or physical therapist, 2) presence of pain with duration of symptoms > 24 h, 3) decreased running mileage, or 4) missed workouts. Lower extremity was defined as any anatomic structure caudal to the lumbar spine. Included studies had to report on RRI. We excluded studies that did not mention clinical assessments, as well as studies using 3D analysis (camera/video) for interpretation. We excluded studies investigating 3D running kinematics (3D biomechanical risk factors) as this review focused on factors evaluated by clinicians. Due to time and expense, 3D is not regularly used by clinicians. We also excluded 2D video analysis as the validity and reliability of this evaluative method is still being established and the focus of this review was objective assessments that are frequently used by clinicians [25–27]. We also excluded military studies as the running conditions (e.g. footwear, carrying load, clothing) are usually different from recreational or competitive runners that would be seen in a community-based setting. Our inclusion criteria allowed for a variety of runner characteristics and follow-up points.

### Study selection

Two authors (SC and JM) reviewed abstracts and selected full text articles independently. Disagreements on whether to include an article were resolved by consulting a third author (CC).

### Data extraction

Data regarding study population (e.g., gender), definition of injury, clinical assessment measure investigated, strength of association statistics, methodological quality of studies and overall quality of the evidence were extracted from full text articles by one reviewer (SC), and confirmed by a second reviewer (JM). Included studies presented all needed data in the manuscript; therefore, no authors were contacted for further information.

### Quality of studies

Included full text articles were each assessed independently by two authors (SC and JM) using the Critical Appraisal Tool (CAT), adapted form of the *Critical Appraisal Form for Quantitative Studies* to evaluate the methodological quality of the selected papers [28, 29]. This tool was chosen because a similar study investigating biomechanical risk factors in runners with defined injuries also used the adapted CAT [29]. The tool is designed to evaluate study quality based on the sample, measures, methods, and outcomes. Items that met criteria, ‘+’, were added to the total score, with the best quality score of 16. A CAT score of > 75% was deemed good quality, 50–75% moderate quality, and lower than 50% poor quality [29].

To evaluate the overall quality of evidence and strength of the findings for of the each clinical assessment alteration category, the GRADE approach (Grading of Recommendations Assessment, Development and Evaluation) [30] was used. The quality of each specific clinical assessment alteration category (Low or very low, as these were observational studies) was based on the performance of the studies against five domains: Risk of bias (methodological quality of each clinical assessment test alteration) [31], inconsistency (heterogeneity within assessment test categories) [32], indirectness (applicability of the findings in terms of population and outcomes) [33], imprecision (the number of participants and events and width of confidence level for each assessment) [34], and publication bias (the probability of selective publication) [35].

## Results

### Search results

**Fig. 1:**
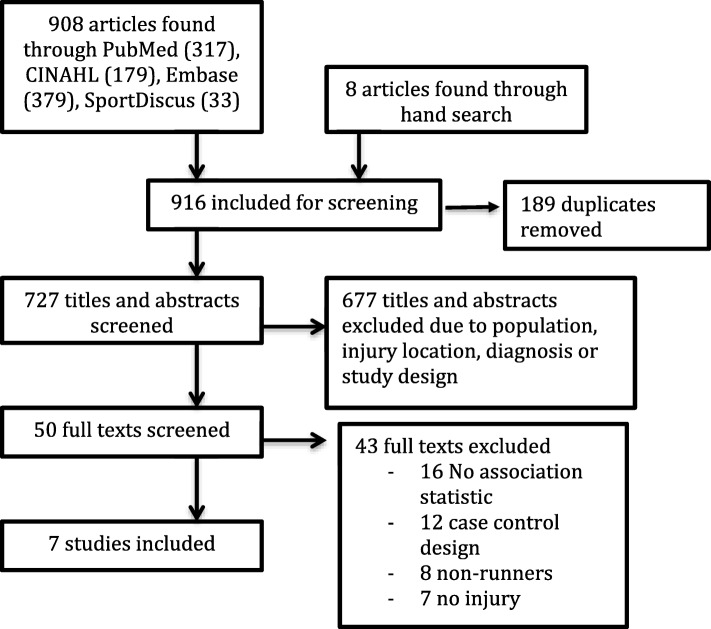
PRISMA flow diagram of studies in systematic review

Initially, before 189 duplicates were removed, the search yielded 916 results (PubMed 317, Embase 379, SPORTDiscus 33, CINAHL 179, and 8 via hand search)(Fig. 1). After the first screening, 50 full-text articles were retrieved. Following a consensus meeting, seven articles were included in this review. Reference checking did not find any additional studies.

A Patient, Exposure, Outcomes (PEO) table, which describes attributes of each study (author, population, exposure, and injury definition) is included in Appendix 2. Descriptions of the objective musculoskeletal clinical assessments identified in the included studies and their methods of measurement have been outlined in Appendix 2. The number of runners included in each study sample ranged from 59 to 532.

### Quality of studies

The results of the assessment of quality of each study using the critical appraisal tool are reported in Table 1.

**Table 1:** Quality assessment of included studies – adapted from the Critical Appraisal Form (CAT) for Quantitative Studies [28, 29]

Author	I-1	I- 2	I-3	I-4	I-5	I- 6	I- 7	I-8	I-9	I-10	I-11	I-12	I-13	I-14	I-15	I-16	T.S	T.%
Buist et al., 2010 [36]	+	+	–	+	+	+	–	+	+	–	+	+	+	+	+	+	13	81.25
Finnoff et al., 2011 [39]	+	+	–	+	+	+	–	+	+	–	–	+	+	+	+	+	12	75.0
Hespanhol Junior et al., 2016 [16]	+	+	–	+	+	+	–	+	+	–	–	+	+	+	+	+	12	75.0
Luedke et al., 2015 [38]	+	+	–	+	+	–	+	+	+	+	–	+	–	+	–	+	11	68.75
Plisky et al., 2007 [37]	+	+	–	+	+	+	+	+	+	+	+	+	+	+	+	+	15	93.75
Ramskov et al., 2013 [41]	+	+	–	–	+	+	–	+	+	–	–	+	+	+	+	+	11	68.75
Yagi et al., 2013 [40]	+	+	–	+	+	+	–	+	+	–	–	+	+	+	+	+	12	75.0

Note. Item 1: Purpose of the study was clearly stated, Item 2: Study design was appropriate, Item 3: Study detected sample bias, Item 4: Measurement biases were detected in the study, Item 5: Sample size was stated, Item 6: The sample was described in detail, Item 7: Sample size was justified, Item 8: Outcomes were clearly stated and relevant, Item 9: Method of measurement was described sufficiently, Item 10: The measures used were reliable, Item 11: The measures used were valid, Item 12: The results were reported in terms of statistical significance, Item 13: The analysis methods used were appropriate, Item 14: Clinical importance was reported, Item 15: Missing data were reported when appropriate, Item 16: Conclusions were relevant and appropriate given methods and results of the study

Abbreviations I- Item, T.S- total score, T%- total CAT %, meets criteria ‘+’, does not meet criteria ‘-’

Among the seven studies included in this review, per the CAT, two were of good methodological quality (> 75%) [36, 37] and five were of moderate quality (50–75%) [16, 38–41]. The majority of methodological shortcomings were observed in the following items: sample bias (7/7 studies) [16, 36–41], reporting validity of measures (5/7 studies) [16, 38–41], justification of sample size (5/7 studies) [16, 38–41], and reporting reliability of measures (5/7 studies) [16, 38–41].

The included studies in this review were all observational design, and therefore per the GRADE approach were considered of low quality of evidence overall [31]. When evaluating each domain, the clinical assessment alterations categories were downgraded either for imprecision, indirectness, inconsistency or all three, resulting in very low quality evidence for each clinical assessment alteration investigated in this review [33, 34, 42]. Publication bias refers to the probability of selective publishing and due to the limited amount of studies for each the clinical assessment alterations(up to three) this item was not used to downgrade evidence in this review [35]. The results of GRADE are reported in Table 2.

**Table 2:** Clinical measures and the reported predictive statistics in the 7 studies investigated in this review

Author, year	Statistical Analysis	Assessment Method	Values (uninjured)	Values (injured)	Association Statistic, 95% Confidence Interval; *p*-value
**Hip Strength**					
**Hip abduction** (GRADE- Very low +++O) ^b,c,d^					
Finnoff et al., 2011 [39]	Bivariable logistic regression	(%BWxheight) = Torque(Nxm)× 100/[BW(N)x height(m)]	2.57(0.53)%	3.14(0.63)%	**OR:5.35, 95% CI= 1.46, 19.53;** ***p*** **:<.01**
Luedke et al., 2015 [38]	Bivariable logistic regression	Force (N)x resistance moment arm (m)/body mass (kg).	Boys: R = 0.25(0.07) Nm/Kg L = 0.25(0.08) Nm/Kg Girls: R = 0.25(0.08) Nm/Kg L = 0.26(0.07) Nm/Kg	NR	Boys: Shin pain tertiles Weakest:OR:1.25, 95% C=I 0.2, 9.9. Middle: OR 1.00, NA Girls: Shin pain tertiles Weakest OR:1.23, 95% CI= 0.7, 21.6, Middle: OR 2.28, 95% CI= 0.2, 28.0
**Hip adduction** (GRADE- Very low ++OO)^c,d^					
Finnoff et al., 2011 [39]	Bivariable logistic regression	(%BWxheight) = Torque(Nxm)× 100/[BW(N)x height(m)]	2.79 (0.61)%	2.87 (0.45)%	OR: 1.23, 95% CI= 0.48, 3.17
**Hip abduction to adduction ratio** (GRADE- Very low ++O)^c,d^					
Finnoff et al., 2011 [39]	Bivariable logistic regression	NR	1.12 (0.28)%	1.06 (0.25)%	OR: 14.14, 95% CI= 0.90, 221.06
**Hip internal rotation** (GRADE- Very low ++OO)^c,d^					
Finnoff et al., 2011 [39]	Bivariable logistic regression	(%BWxheight) = Torque(Nxm)× 100/[BW(N)x height(m)]	1.68 (0.40)%	1.88 (0.68)%	OR: 2.75, 95% CI= 0.33, 23.17
**Hip external rotation** (GRADE- Very low ++OO)^c,d^					
Finnoff et al., 2011 [39]	Bivariable logistic regression	(%BWxheight) = Torque(Nxm)× 100/[BW(N)x height(m)]	1.44 (0.31)%	1.34 (0.26)%	OR: 0.35, 95% CI= 0.03, 4.48
**Hip external to internal rotation strength** (GRADE- Very low ++OO)^c,d^					
Finnoff et al., 2011 [39]	Bivariable logistic regression	NR	0.87 (0.17)%	0.74 (0.13)%	**OR: 0.01, 95% CI= < 0.01, 0.44;p:0.02**
**Hip flexion** (GRADE- Very low ++OO)^c,d^					
Finnoff et al., 2011 [39]	Bivariable logistic regression	(%BWxheight) = Torque(Nxm)× 100/[BW(N)x height(m)]	2.84 (0.61)%	2.49 (0.92)%	OR: 0.40, 95% CI= 0.05, 3.09
**Hip extension** (GRADE- Very low ++OO)^c,d^					
Finnoff et al., 2011 [39]	Bivariable logistic regression	(%BWxheight) = Torque(Nxm)× 100/[BW(N)x height(m)]	3.15 (0.79)%	2.87 (0.79)%	OR: 0.64, 95% CI= 0.21, 1.90
**Hip flexion to extension strength** (GRADE- Very low ++OO)^c,d^					
Finnoff et al., 2011 [39]	Bivariable logistic regression	NR	0.86 (0.15)%	0.96 (0.13)%	OR: 0.17, 95% CI= 0.021, 5.61
**Hip Range of Motion**					
**Hip IR ROM** (GRADE- Very low ++OO)^b,c^					
Buist et al., 2010 [36]	Multivariable logistic regression	Goniometer	Male L = 30.6(8.1)° R = 31.1(8.8)° Female L = 35.9(9.5)° R = 37.7(8.3)°	NR	Male: HR: 1.00 **Female HR 0.98** **aHR: 0.99, 95% CI= 0.97, 1.01; P:0.08**
Yagi et al., 2013 [40]	Multivariable logistic regression	Goniometer	Male: 12.4 (8.7)° Female: 25.5 (9.5)°	Male: MTSS:12.9(5.8)° SF: 7.5 (3.5)° Female: MTSS: 31.1 (9.9)° SF: 20.7(7.6)°	Male MTSS: aOR: 0.99, 95% CI 0.91, 1.08 SF: aOR: 1.26, 95% CI 0.81, 1.96 Female MTSS: **aOR 0.91, 95% CI 0.85, 0.99; p:0.02** SF: aOR:1.00, 95% CI 0.88, 1.12
*Hip ER ROM* (GRADE- Very low ++OO)^b,c^					
Buist et al., 2010 [36]	Multivariable logistic regression	Goniometer	Male: L = 39.7(11.6)° R = 40.2(12.9)° Female L = 45.7(14.3)° R = 45.8(13.9)°	NR	Male: HR: 1.01 Female: HR:1.00
Yagi et al., 2013 [40]	Multivariable logistic regression	Goniometer	Male: 39.7(8.8)° Female: 35.1 (9.0)°	Male: MTSS: 44.5(8.9)° SF: 40.0(14.1)° Female: MTSS: 37.4 (8.5)° SF: 43.3 (2.9)°	Male: MTSS: aOR: 0.96, 95% CI 0.88, 1.03 SF: aOR: 0.76, 95% CI 0.56, 1.03 Female MTSS: aOR:1.0, 95% CI 0.93, 1.08 SF: aOR:1.0, 95% CI 0.90, 1.11
**Hip Alignment**					
*Q angle* (GRADE- Very low ++OO)^b,c^					
Hespanhol junior et al., 2016 [16]	Multivariable logistic regression	Goniometer	10.1(5.1)°	11.8(5.0)°	OR:0.9, 95% CI= 0.8, 1.0
Ramskov et al., 2013 [41]	Bivariable logistic regression	Goniometer	L = 11.1(4.4)° R = 11.1(5.0)°	L = 8.2(4.5)° R = 9.1(4.5)°	cRR: 1.26, 95% CI= 0.49, 3.23
*Leg length* (GRADE- Very low +OOO)^d^					
Hespanhol junior et al., 2016 [16]	Multivariable logistic regression	Measuring Tape	0.5(0.6)cm	0.4(0.6)cm	OR: 1.3, 95% CI= 0.6, 2.7
**Hip Flexibility**					
*Straight leg raise* (GRADE- Very low ++OO)^c,d^					
Yagi et al., 2013 [40]	Multivariable logistic regression	Goniometer	Male:74.3(10.4)° Female:76.1 (12.5)°	Male: MTSS:77.6(8.5)° SF:60.0 (14.1)° Female: MTSS:77.7(11.0)° SF:78.3 (7.6)°	Male MTSS: aOR: 0.99, 95% CI= 0.60, 1.29 SF: aOR: 1.38, 95% CI= 1.04, 1.83 Female MTSS: aOR: 0.98, 95% CI= 0.92, 1.05 SF: aOR:1.00, 95% CI= 0.90, 1.11
**Knee Strength**					
**Quadriceps strength** (GRADE- Very low ++OO)^c,d^					
Luedke et al., 2015 [38]	Bivariable logistic regression	Force (N)x resistance moment arm (m)/body mass (kg).	Boys: R = 0.31(0.06)Nm/kg L = 0.30(0.05)Nm/kg Girls: R = 0.28(0.04)Nm/kg L = 0.28(0.05)Nm/kg	NR	Boys: Shin pain Tertiles Weakest OR:0.83, 95% CI= 0.1, 6.1 Middle: No injured Girls: NR
**Hamstring strength** (GRADE- Very low ++OO)^c,d^					
Luedke et al., 2015 [38]	Bivariable logistic regression	Force (N)x resistance moment arm (m)/body mass (kg).	Boys: R = 0.22(0.06) Nm/kg L = 0.21(0.06) Nm/kg Girls: R = 0.20(0.03) Nm/kg L = 0.20(0.04) Nm/kg	NR	Boys: Shin pain Tertiles Weakest OR:1.20, 95% CI= 0.2, 8.8, Middle: OR: 0.40, 95% CI= 0.1, 5.2 Girls: Shin pain Tertiles Weakest: OR: 1.33, 95% CI= 0.2,16.7 Middle: OR: 0.55, 95% CI= 0.1, 9.9
**Ankle Alignment**					
**Navicular drop** (GRADE- Very low ++OO)^b,c^					
Buist et al., 2010 [36]	Multivariable logistic regression	NR	Male: L = 6.6(3.5)mm R = 6.7(3.5)mm Female: L = 6.0(3.1)mm R = 6.2(2.8)mm	NR	Male HR 1.02 **Female HR 0.92** **aHR- 0.87, 95% CI= 0.77, 0.98; p:0.01**
Plisky et al., 2007 [37]	Bivariable logistic regression	Ruler perpendicular to the floor	> 10 mm N Boys: 20(43.5) N Girls:24(40.7) < 10 mm N Boys:26(56.5) N Girls:25(59.3)	N 15.8 N 14.9	OR: 1.0 OR: 0.9, 95% CI= 0.3, 2.8
Yagi et al., 2013 [40]	Multivariable logistic regression	Goniometer	Male: 4.5(3.4)mm Female:4.2(2.4)mm	Male MTSS:4.9(3.0) mm SF: 2.4(3.1)mm Female MTSS:4.9(3.0)mm SF: 3.4(2.9)mm	Male MTSS: aOR:0.93, 95% CI= 0.75, 1.14 SF: aOR: 1.00, 95% CI= 0.71, 1.42 Female MTSS: aOR: 0.90, 95% CI= 0.70, 1.19 SF: aOR: 1.5, 95% CI= 0.95, 2.51
**Foot posture index** (GRADE- Very low ++OO)^c,d^					
Ramskov et al., 2013 [41]	Bivariable logistic regression	Method by Redmond et al	N: Very pronated:1 Pronated: 14 Neutral: 79 Supinated: 0 Very supinated: 0	N: Very pronated:3 Pronated: 4 Neutral: 14 Supinated: 0 Very supinated: 0	cRR: 1.65, 95% CI= 0.65, 4.17
**Ankle Range of Motion**					
**Ankle dorsiflexion** (GRADE- Very low +OOO)^c^					
Buist et al., 2010 [36]	Multivariable logistic regression	Goniometer	Male: L= KB- 104.7(7.8)° KS-99.2(8.2)° R= KB-104.6(7.5)° KS-99.2(7.8)° Female: L= KB-103.6(11.5)° KS-99.0(10.9)° R= KB-103.8(8.7)° KS- 99.1(9.2)°	NR	Male HR: 1.01(KB) HR: 1.01 (KS) Female HR: 1.00(KB) HR: 1.00 (KS)

*OR* odds ratio, *aOR* adjusted odds ratio, *HR* Hazard ration, *aHR* adjusted hazard ratio, *RR* risk ratio, *cRR* cumulative relative risk, *SF* stress fracture, *MTSS* medial tibial stress syndrome, *KB* knee bent, *KS* knee straight

GRADE working group grades of evidence: (bolded? heading for below items)

Low quality: Further research is likely to have an important impact on our findings

Very low quality: We are uncertain about the findings

a. Item was downgraded due to risk of bias in methods, recruitment, follow up or selective reporting

b. Item was downgrade due to inconsistency such as difference in measurement method, population, injury definition within the studies included in the outcome

c. Item was downgraded due to indirectness and therefore applicability of findings regarding population or outcomes

d. Item was downgraded due to imprecision (i.e. small sample size < 300)

### Objective musculoskeletal clinical assessments (Table 2)

#### Hip strength

Evidence for hip strength was of very low quality (hip abduction strength downgraded due to indirectness, inconsistency, and imprecision whereas the rest were downgraded due to indirectness and imprecision). Of the two studies investigating hip abduction strength, one study [39] reported that stronger hip abduction strength was significantly associated with injured runners (OR = 5.35, 95% CI= 1.46, 19.53**)** whereas the other study [38] found no significant association. Finnoff et al. [39], also reported a significant protective association with increased hip external rotation to internal rotation strength ratio RRI (OR = 0.01, 95% CI= < 0.01, 0.44). There were no significant associations between hip adduction, abduction to adduction ratio, external rotation, internal rotation, flexion, extension, flexion-to-extension strength ratio and RRI [39].

#### Hip joint range of motion

Evidence for hip joint range of motion was of very low quality (downgraded due to indirectness and inconsistency). Two studies [36, 40] investigated hip internal and external range of motion, of which one study [40] found that increased hip internal rotation was protective against RRI in females that developed medial tibial stress syndrome (aOR = 0.91, 95% CI= 0.85, 0.99) [40].

#### Hip alignment

Evidence for hip alignment was of very low quality (Q angle downgraded for indirectness and inconsistency, and leg length downgraded for imprecision). Two studies [16, 40] investigated Q angle and one study [16] investigated leg length. The studies were unable to find significant relationships between hip alignment tests investigated and RRI.

#### Hip flexibility

Evidence for hip flexibility was of very low quality (downgraded for indirectness and imprecision). One study [40] investigated straight leg raise and did not find significant association between straight leg raise test and RRI.

#### Knee strength

Evidence for knee strength was of very low quality (downgraded for indirectness and imprecision). One study [38] investigated knee strength using a HHD and did not find a significant association between quadriceps strength or hamstring strength and RRI.

#### Ankle alignment

Evidence for ankle alignment was of very low quality (navicular drop downgraded for indirectness and inconsistency, and foot posture index downgraded for indirectness and imprecision). Three studies [36, 37, 40] investigated navicular drop and the development of running injuries. One study [36] found a significant protective relationship between decreased navicular drop amount in females and injury (HR = 0.92); two studies did not find a significant relationship between navicular drop and injured runners. One study [41] investigated the Foot Posture Index [43] and did not find a significant relationship between foot posture and injured runners.

#### Ankle joint range of motion

Evidence for ankle range of motion was of very low quality (downgraded for indirectness). One study [36] investigated ankle dorsiflexion range of motion and did not report a significant association between ankle dorsiflexion (in knee straight and bent) and RRI.

## Discussion

### Findings within the studies

The goal of this study was to summarize the results of stand-alone studies that have investigated clinical assessment and risk of injury. Synthesizing the work should improve an understanding of which factors may be transferable to a clinical environment. Stand-alone findings such as increased hip external to internal rotation strength ratio and decreased navicular drop were protective of injury, but only in a few studies. We also found that increased hip abduction strength was predictive of injury and decreased hip internal rotation was protective of injury in runners, largely contradicting clinical thought and results from non-longitudinal studies of association [44]. In no cases did we find compelling evidence from multiple studies of common predictors of injury risk in running. Also, all clinical assessment alteration categories had very low quality of evidence; therefore, clinicians should be very cautious interpreting the results below.

As stated, increased hip external to internal strength ratio was seen to be protective for injury in runners that developed patella femoral pain syndrome. This finding was reported in one study by Finnoff et al. [39] Although the authors did not operationally define this ratio, it is assumed that an increase in hip external rotator strength when compared to internal rotator strength would be protective for runners. The hip external rotators muscles control femoral internal rotation and a lack of control may be linked with running injury [45, 46]. It is important to note there were several confounders in this study. The study did not report running distance per week (mileage) nor did it report any injury history, both of which have been associated as risk factors for injury. Because these athletes were high school runners, these factors could have significantly influenced results [1].

Decreased navicular drop was seen to be protective of injury in this review. This finding was reported in one study [36]; however, it was not significant among the two other studies [25, 28] that did investigate this measure. Excessive pronation of the foot causes tibial rotation and has been seen to be related to medial stress syndrome in runners [47]. This finding was investigated in novice runners participating in a 13-week training program for a 4-mile running event and therefore cannot be applied to all running populations in general.

Increased hip abduction strength was found to be predictive of injury in one cohort study. The finding that runners with stronger hip abductors were more associated with RRI may have been due to a number of confounders. The participants included in the study were high school athletes, possibly novice runners. As mentioned before, weekly training mileage and injury history were not reported. Finnoff et al. [39], theorized that the injured subjects in the group had higher body mass index (BMI), which could have led to higher hip abduction moments. To compensate for these larger moments, the runners may have developed increased hip abductor (eccentric) strength over time [39]. This finding shows that some injured runners may have increased strength, specifically if they are younger or novice runners with a higher BMI. Caution should be used when interpreting this result with all running populations.

Decreased hip internal rotation was found to be protective in one cohort study [40]. Excessive hip internal rotation has been associated with injury during jump landing tasks and lack of control of the lower extremity in the frontal and transverse planes has also been hypothesized as a cause for injury in runners [48, 49]. Decreased mobility could therefore be beneficial and protective for runners, as it would require less neuromuscular control. This finding shows that stiffness in runners may not be an impairment as previously thought [50, 51], specifically if they are young and may not have developed the neuromuscular control needed to stabilize the limb. Caution should be used while interpreting the findings of this study as participants were high school runners. Shin pain was the only injury reported. Mileage of the runners was not reported; however, frequency of training was. Experience was noted as national, state, or entry level, however no history of running injury or amount of running miles was reported.

### Findings between the studies

The GRADE level of evidence quality was very low for all objective assessment alteration categories included in this review. Studies were downgraded for either indirectness, inconsistency, imprecision or all three. There were no common predictors across a number of studies in this review. There may be several reasons for the lack of commonality or the occasional findings that are contradictory to clinical thought, such as differences in subject demographics, different measurement methods, measurement variability within clinical assessments, inconsistent definitions of injury and runner, different statistical models, and study bias. These issues have been further addressed below.

There were a wide range of different assessments used to compare clinical assessment alterations and future injury within the seven prospective studies, and studies used different methods when measuring the same construct. For example, ankle alignment was measured with navicular drop [36, 37, 40] or Foot Posture Index [41]. This lack of homogeneity between studies resulted in difficulties comparing clinical assessments between studies, even when studies focused on a similar construct (e.g., alignment).

A variety of methods was used to define and report the clinical assessments, even when the same testing device was used. For instance, weakness in hip HHD assessment was often reported by asymmetry between left and right sides [39, 40]. However, another study [38] divided strength into three tertiles (weakest, middle and strong) across participants and used the strongest strength values as the comparator. One study [38] multiplied the HHD reading by the moment arm and then normalized it to the participant’s body mass. The other studies normalized HHD values to body mass and height [39]. This variability in the reporting of muscle strength assessments made it difficult to compare studies, perform meta-analyses, or identify common patterns of muscle strength in included prospective studies.

Population and injury definitions were also heterogeneous among studies. Running populations in studies varied from novice to recreational, with more males than females in the Q angle studies [13, 29]. Running related injury has been defined many ways in the literature, as evidenced by the wide variability of injury incidence rates reported in various studies [1, 2, 52]. When defining an injury, studies used: 1) evaluation by a medical physician, athletic trainer or physical therapist [39], 2) presence of pain with duration of symptoms > 24 h [37], 3) decrease in running mileage, 4) missed workouts [16] or, 5) a combination of the variables listed [36, 38, 40, 41] all which were included in our study. Consistent reporting about injury severity, the course of treatment, previous injury, or whether the runner had sought assistance from a health care provider was lacking. Difference in levels of injury severity would likely alter associational modeling and influence the statistical significance of the findings.

Lastly, statistical modeling was different among studies. Three studies used a multivariable model, whereas four studies used a bivariable model. Among the three studies that used a multivariable model, measures of independent variables such as age [36], other clinical tests [16] and BMI [40] were also included in the regression analysis model. This could have influenced the relationship between singular clinical test (such as navicular drop) [36] and RRI.

Previous reviews investigating the risk of RRI have also reported similar criticisms [53, 54]. Winter et al. [53] investigated fatigue and RRI, and were unable to find conclusive patterns of associations due to a lack of homogeneity of the runners, small sample sizes, and the distances that were run to determine fatigue. A systematic review studying vertical ground reaction force and injury was also unable to make recommendations due to a lack of prospective studies investigating this variable and its association with injury [54]. When reviewing biomechanical risk factors, Aderem and colleagues [29] concluded that shod female runners with iliotibial band syndrome (ITBS) may have associated increased peak knee internal rotation and peak hip adduction during stance (based on one prospective cohort study), but because of limitations in effect size and the number of studies and methods, the authors did not make any additional recommendations. In the one review that investigated alterations to the musculoskeletal system, similar to the current study, i.e., plantar pressures, the authors concluded there was inconsistency among studies and suggested improved methodology for future research [55].

### Limitations

There are several limitations to this review. Studies with post-operative populations were excluded from the study, so it is possible the runners included in the selected studies had less severe injuries, which potentially influenced the clinical assessment alterations between baseline and future injury. This was performed to better generalize the results to the population of runners commonly seen in outpatient community-based clinics, who often present without having seen a surgeon [56].

## Conclusion

This review suggests that objective assessments that measure alterations in muscle strength, flexibility, alignment, and range of motion of the lower extremity had very low quality of evidence. Within the studies there were several confounders such as participant’s experience, unknown injury history, and unknown weekly running mileage, all of which have been seen to be associated with RRI [1]. Among the studies, there were a limited number of studies investigating each assessment, inconsistent results, different measurement methods among studies, measurement variability within clinical assessments, inconsistent definitions of injury and runner, different statistical modeling, and study bias. Future studies should aim to improve the quality of the studies as well as use standardized assessments and minimize confounders when conducting clinical research to predict injury in runners.

## Appendix 1

**Search terms used in PubMed database**

Injury[tiab] OR Injuries[tiab] OR “physiopathology” [Subheading] OR “injuries” [Subheading] OR “Wounds and Injuries”[Mesh]) AND (Runner[tiab] OR Runners[tiab] OR Running) AND (Muscle Strength OR Muscle Weakness OR Strength[tiab] OR Weakness[tiab]) AND (sensitive[tiab] OR sensitivity[tiab] OR specificity[tiab] OR sensitivity and specificity[MeSH] OR diagnosis[tiab] OR diagnostic[tiab] OR diagnosed[tiab] OR diagnosis[MeSH] OR diagnosis[sh] OR cross-sectional studies[Mesh] OR cross-sectional[tiab]) NOT (review[ptyp] OR Editorial[ptyp] OR Letter[ptyp] OR Case Reports[ptyp] OR Comment[ptyp]) NOT (animals[mh] NOT humans[mh]

## Appendix 2

**Table 3:** PEO (Population, Exposure, Observation) Table; description of included articles

Author, Year of publication	Population N (gender) Follow up	Exposure (Clinical Measure)	Observation (Injury Definition)
Buist et al., 2010 [36]	532 novice runners (226 male, 306 female); 8 or 13-week program	Range of motion with universal goniometer: Internal and external ROM of the hip: assessed in supine and the tested hip and knee flexed to 90° Ankle dorsal flexion- measured both with the knee fully extended and flexed to 90° passively, in supine position. Alignment: Navicular drop- assessed by measuring the change in the height of the navicular tuberosity between sitting with the subtalar joint in neutral position and standing, weight-bearing with the subtalar joint in relaxed stance, measurements were made twice for each foot, results were averaged	Self-reported musculoskeletal pain of the lower extremity or back causing a restriction of running for at least 1 week, i.e. 3 scheduled consecutive training sessions.
Finnoff et al., 2011 [39]	98 high school cross country and track athletes (53 male and 45 female); Cross country and/or track season	Leg Length- measuring from anterior superior iliac spine (ASIS) to a point 2 cm proximal to the apex of medial malleolus Muscle strength with HHD for break test: Hip flexion- seated hip flexion to 120° with HHD on distal aspect of thigh Hip Extension- extend test hip to a neutral position with the knee extended while maintaining neutral hip rotation with HHD against the subject’s posterior calcaneus Hip External Rotation- seated knees were also flexed 90° with the hip in neutral rotation with HHD positioned 2 cm proximal to the apex of the medial malleolus Hip Internal rotation- position identical to the one used for hip external rotation strength testing with HHD positioned 2 cm proximal to the apex of the lateral malleolus Hip Abduction- 30° abduction with neutral hip flexion, extension, rotation) HHD positioned 2 cm proximal to the apex of lateral malleolus Hip Adduction- neutral flexion, extension, rotation (subject allowed to grasp table for trunk stability). Strength test was performed with the HHD placed 2 cm proximal to the medial malleolus Pain- Visual Analogue Scale (10 cm)	ATC monitored and evaluated by physician investigators: ITBS suspected with lateral knee pain, local tenderness over lateral knee where ITB crosses over condyle, exacerbated by flexion and extension while applying pressure PFP suspected with anterior knee pain, exacerbated by deep knee flexion and/or climbing stairs, and by reproduction of pain with at least one of following: 1) pressure over distal quadriceps with active contraction and 2) direct palpation of medial and lateral patellar facets
Hespanhol Junior et al., 2016 [16]	89 recreational runners (68 male/21 female); 12 weeks	Leg Length: in a supine position, lower limbs relaxed. Measuring tape was used to determine the real length of the lower limbs i.e., the length between the ASIS of the hemipelvis to the center of the ipsilateral medial malleolus of both lower limbs. The lower limb length discrepancy was considered normal when lower than 1.0 cm Q-angle: In sports clothes and standing barefoot in an orthostatic position. A straight line was traced using a ruler from the ASIS to the center of the patella, and a second line was traced from the center of the patella to the tibial tuberosity. The angle formed by the intersection of these two lines constitutes the Q-angle, which was measured by a universal goniometer. Values between 10° and 15° were considered normal for both genders	Missed at least one training session due to musculoskeletal pain (Biweekly questionnaire reporting musculoskeletal pain, number of training sessions missed, pain intensity (10 point numerical pain rating scale), description (type and anatomical location) of new injury)
Luedke et al., 2015 [38]	68 High school runners (16 male, 47 female); Interscholastic cross-country season	Muscle strength with HHD for bilateral peak isometric strength (2 trials): Hip abduction- sidelying, non-test limb was positioned in 30–45° of hip flexion and 90° of knee flexion, pelvis was stabilized to the table using a strap, test hip was in 0° of extension and abducted to parallel with the table and HHD was placed just proximal to the lateral malleolus on the test limb Knee Extension: seated at the end of a table with the test knee at 45° of flexion, stabilizing strap was placed around the thighs and table, resistance applied to the anterior aspect of the tibia 5 cm proximal to the ankle joint Knee Flexion - prone and the test knee flexed to 45°, stabilizing strap was placed around the pelvis and table with resistance applied to the posterior aspect of the tibia 5 cm proximal to the ankle joint	Injury- required athlete to be removed from practice or competitive event, or miss a subsequent practice/competitive event PT or LAT determine injury: Knee pain 1. Pain around ant aspect of knee 2) insidious onset 3) no incidence of trauma Shin injury 1) continuous or intermittent shin pain 2) exacerbated by weight bearing activities 3) local pain with palpation along tibia
Plisky et al., 2007 [37]	105 high school cross country runners (59 male, 46 female); 13 week cross country season	Alignment: Navicular drop (normalized to full foot length and truncated foot length) - in unilateral standing position, the runner’s foot placed subtalar neutral, ruler was placed next to the medial foot perpendicular to the floor and was read (mm) at the height of the navicular tubercle, 2 measurements were recorded, relaxing in between, and the difference value was documented as navicular drop (Runners were allowed to maintain their balance by placing a hand on a handrail during unilateral stance)	PT and ATC examined runner for MTSS criteria 1) continuous or intermittent pain in the tibial region, exacerbated by weight bearing activities 2) local pain with palpation along distal 2/3 of posterior medial tibia
Ramskov et al., 2013 [41]	59 novice runners (31 male, 28 female); 10 weeks	Alignment: Foot Posture Index [43]. Q angle- center of the goniometer placed upon the middle of the patella, one arm of the goniometer placed along the line connecting ASIS with the middle of patella, other arm was placed along the line connecting the middle of patella and the tibial tuberosity	Injury: Any running-related injury to lower extremity or lower back that causes at least one week of restricted running Diagnoses by physiotherapist ~ 1 week after injury; if extensive exam needed referred to university hospital medical center division of sports traumatology
Yagi et al., 2013 [40]	230 high school runners (134 male, 96 females); 3 years	Range of motion: Hip rotation- measured with the hip and knee flexed at 90° in the sitting position; the hip and knee were rotated internally and externally to firm end feel with the angles relative to the initial position. Ankle dorsiflexion-measure in two positions with knee in extension and 90° flexion; ankle was passively moved into dorsiflexion from a neutral-starting position until a firm end feel was elicited (examiner first identified the neutral position of the subtalar joint and then kept the neutral position while dorsiflexing the foot until a firm end point was felt) Flexibility: Straight leg raising – supine, passively into hip flexion until firm resistance was felt and the pelvis tilted posteriorly Alignment (knee varus or valgus and ankle eversion inversion in standing closed feet), Navicular drop test-distance between the navicular tuberosity and the floor during [1] quiet tandem stance with the subtalar joint placed in neutral, and no load on the foot, and [2] relaxed tandem stance with full load on the foot Q angle- center axis of a long-arm goniometer placed over the center of the patella, proximal tibia was palpated, and the lower goniometer arm was aligned along the patellar tendon to the tibial tubercle, upper arm of the goniometer was pointed directly at the anterior superior iliac spine Strength: Hip abduction isometric break test with HHD	Could not run for 7 days due to shin pain - radiographs taken (if reinjured counted in study as additional subject) and diagnosis by sports physician

*NR* not reported, *m/wk*. miles per week, *yr*. year, *ROM* range of motion, *HHD* Hand held dynamometer, *MTSS* medial tibial stress syndrome, *SF* stress fracture

## Abbreviations

aOR: Adjusted odds ratio
aRR: Adjusted risk ratio
HHD: Hand held Dynamometer
HR: Hazard ratio
OR: Odds ratio
RR: Risk Ratio
RRI: Running-related Injury
